# Heredity and the Question of Forced Labour

**Published:** 1903-03-14

**Authors:** 


### HEREDITY AND THE QUESTION OF FORCED LABOUR.

The labour problem with which we are confronted in South Africa and the difficulty experienced in inducing the natives to undertake regular work bring into prominence the fact that one of the main causes of that heavy responsibility in regard to subject races which has come to be spoken of as "the white man's burden" arises from their hereditary tendency, their long-ingrained instinct to remain contented with small things.

It is the fashion nowadays to believe in the influence of heredity; but if there is anything in heredity at all, surely it is out of the question to expect, as some people seem to do, that races which, as far back as history will take us, and probably as far back as from the first differentiation of man, have led a savage life should all at once, and in defiance of the temptations cast in their way by the white man, show themselves fitted for the freedom and responsibilities of civilisation. Thus are we brought face to face with the question how far may we go in controlling and regulating the labour of the blacks without having the word "slavery" cast in our teeth, and being taunted with coquetting with that unclean thing; for all experience seems to point to the probability that we shall have for many years, nay for many generations, to accept the necessity for differential treatment between blacks and whites. In regard to this problem it may be worth while to recall the miserable position still occupied by the mass of the negroes in the United States even forty years after complete emancipation, and after contact with, and a certain amount of interbreeding with, white races for a couple of centuries. Dr. Edelman, of Alabama,[1] says that there are in the United States 866 criminals to every million among the white inhabitants, while there are 2,974 criminals to every million among the negro inhabitants. In Washington, with a population of 277,782 in 1901, 26,062 arrests were made; of these 12,582 were from a white population numbering 189,457, and 13,780 from a coloured population numbering 88,325, and he gives similar figures from a number of Southern towns all showing the enormous preponderance of crime among the negro population. Lest it should be said that in the Southern States the courts are prejudiced against the negro criminal, he adds that in the State of Pennsylvania, where there is little opportunity to assert that any such prejudice exists, the negro furnishes 16 per cent. of the male and 34 per cent. of the female prisoners, though he forms only 2 per cent. of the population; while in Chicago, which is called the "negro heaven," he furnishes 10 per cent. of the arrests, though he forms only $1\frac{1}{3}$ per cent. of the population. In view of this condition of affairs it is being seen in America as we see in South Africa that the only safety lies in work. "In labour lies the redemption of the negro. There are too many negro idlers," we are told, "who are living from the white man's kitchen." In the States we find a demand arising for the strict sanitary supervision of the negro and his habitations, so as to minimise the obvious dangers to the health of the community which arise from negro indifference to all hygienic precautions; for the enforcement of strict vagrancy laws; for the establishment of reformatory schools for the young; and for the creation of large State farms where the negro idler or the negro criminal may be made to work.

Hereditary contentment with small things is, however, at the root of the mischief, and a healthy ambition is a difficult plant to raise, and one which it will take generations to establish among people whom one can make thoroughly happy by a present of offal, as is the case with many in South Africa. It is evident that the white races in South Africa, surrounded as they are by blacks who outnumber them five times over, must take some action and must invent some substitute for the harsh rule of the Boers if this is to be abolished. Even if we regard the sanitary question alone it is impossible to believe that South Africa will long retain its reputation for health if the blacks are allowed to live and to grow up on every side in enormous numbers, according to their old traditions, and to surround every farm and every town with a ring of insanitary abominations. No white race is likely to remain healthy in the midst of an overwhelming multitude of blacks who remain outside the pale in regard to sanitation. Whether then we regard the matter as one of health, education, temperance, progress, or even safety we feel that, however much our humanitarian instincts may tempt us to the contrary, the teachings derived from a study of heredity will have to be obeyed and the negro will have to be governed with a strong hand for many a year to come.

[1] New York Medical News, Jan. 31.

